# Supplementary material for: Comparison of devices used to measure blood pressure, grip strength and lung function: A randomised cross-over study
Source: PLoS One. 2023 Dec 27;18(12):e0289052. doi: 10.1371/journal.pone.0289052 (PMC10752545; doi:10.1371/journal.pone.0289052)
Supplement: S1 Table — (DOCX) [file pone.0289052.s001.docx]

S1 Table: Sample by age group and sex

|  | **45-54** | **55-64** | **65-74** |
| --- | --- | --- | --- |
| **Men** | 18 | 20 | 21 |
| **Women** | 20 | 19 | 20 |
