# Supplementary material for: Comparison of devices used to measure blood pressure, grip strength and lung function: A randomised cross-over study
Source: PLoS One. 2023 Dec 27;18(12):e0289052. doi: 10.1371/journal.pone.0289052 (PMC10752545; doi:10.1371/journal.pone.0289052)
Supplement: S2 Table — (DOCX) [file pone.0289052.s002.docx]

S2 Table: Reliability (intra-cluster correlation ICC) and within-person standard deviation (SD) for each device included in the study

|  | **ICC** | **SD** | **ICC** | **SD** |
| --- | --- | --- | --- | --- |
| **Blood pressure** | **SBP** | | **DBP** | |
| Omron 705-CE | 0.90 | 5.7 mmHg | 0.89 | 3.6 mmHg |
| Omron HEM-907 | 0.91 | 4.9 mmHg | 0.94 | 2.9 mmHg |
| **Grip strength** | **Dominant hand** | | **Non-dominant hand** | |
| Nottingham | 0.96 | 2.4 kg | 0.92 | 3.0 kg |
| Jamar Plus+ Digital | 0.95 | 2.3 kg | 0.93 | 2.4 kg |
| Jamar Hydraulic | 0.96 | 2.0 kg | 0.95 | 2.1 kg |
| Smedley | 0.92 | 2.3 kg | 0.87 | 2.5 kg |
| **Lung function** | **FEV_1_** | | **FVC** | |
| Micro Plus - A&B readings | 0.99 | 80ml | 0.98 | 112ml |
| (All readings) | 0.97 | 108ml | 0.98 | 115ml |
| Easy on-PC - A&B readings | 0.97 | 115ml | 0.98 | 113ml |
| (All readings) | 0.96 | 134ml | 0.97 | 155ml |
