# Supplementary material for: Comparison of devices used to measure blood pressure, grip strength and lung function: A randomised cross-over study
Source: PLoS One. 2023 Dec 27;18(12):e0289052. doi: 10.1371/journal.pone.0289052 (PMC10752545; doi:10.1371/journal.pone.0289052)
Supplement: S3 Table — (DOCX) [file pone.0289052.s003.docx]

S3 Table: Assessment of order effects for all measures

|  | Device (n) | | Independent t-test | | | 95% CI | |
| --- | --- | --- | --- | --- | --- | --- | --- |
|  | A | B | Diff | SE | p-value | Lower | Upper |
| ***SBP, mm Hg (Mean of 2^nd^+3^rd^)*** |  |  |  |  |  |  |  |
| Omron 705 - Omron 907 | 56 | 59 | -2.32 | 1.37 | 0.11 | -5.03 | 0.38 |
| ***DBP, mm Hg (Mean of 2^nd^+3^rd^)*** |  |  |  |  |  |  |  |
| Omron 705 - Omron 907 | 56 | 59 | -0.16 | 1.06 | 0.88 | -2.26 | 1.95 |
| ***Grip strength, kg (max of 4)*** |  |  |  |  |  |  |  |
| Jamar Hydraulic – Smedley | 59 | 59 | -1.18 | 1.03 | 0.26 | -3.23 | 0.87 |
| Nottingham - Jamar Plus+ | 58 | 60 | -1.92 | 1.16 | 0.10 | -4.21 | 0.37 |
| Jamar Plus+ - Jamar Hydraulic | 60 | 58 | -1.18 | 0.60 | 0.05 | -2.37 | 0.01 |
| Jamar Plus+ - Smedley | 59 | 59 | 0.03 | 1.03 | 0.98 | -2.02 | 2.07 |
| Nottingham - Jamar Hydraulic | 57 | 61 | -1.78 | 1.18 | 0.13 | -4.11 | 0.55 |
| Nottingham – Smedley | 60 | 58 | -3.08 | 1.44 | **0.03** | -5.93 | -0.23 |
| ***FEV1, litres (ATS/ERS criteria)*** |  |  |  |  |  |  |  |
| Easy on-PC - Micro Plus | 39 | 35 | -0.02 | 0.03 | 0.53 | -0.08 | 0.04 |
| ***FVC, litres (ATS/ERS criteria)*** |  |  |  |  |  |  |  |
| Easy on-PC - Micro Plus | 35 | 32 | -0.06 | 0.06 | 0.26 | -0.18 | 0.05 |

SE=standard error; CI=confidence interval
