# Supplementary material for: Comparison of devices used to measure blood pressure, grip strength and lung function: A randomised cross-over study
Source: PLoS One. 2023 Dec 27;18(12):e0289052. doi: 10.1371/journal.pone.0289052 (PMC10752545; doi:10.1371/journal.pone.0289052)
Supplement: S4 Table — (DOCX) [file pone.0289052.s004.docx]

S4 Table: Sensitivity analysis for differences in mean and limits of agreement for all measures

|  |  | Independent t-tests | | | 95% CI | | LOA | |
| --- | --- | --- | --- | --- | --- | --- | --- | --- |
|  | N | Diff | SE | p-value | Lower | Upper | Lower | Upper |
| **Blood pressure, mm Hg** |  |  |  |  |  |  |  |  |
| ***SBP: Omron 907 - Omron 705*** |  |  |  |  |  |  |  |  |
| Primary, excluding misordered | 113 | 3.85 | 0.68 | **<0.001** | 2.50 | 5.20 | -10.35 | 18.06 |
| Primary, excluding outliers | 114 | 4.16 | 0.63 | **<0.001** | 2.92 | 5.40 | -8.95 | 17.26 |
| Mean of 3 readings | 115 | 3.92 | 0.64 | **<0.001** | 2.65 | 5.19 | -9.59 | 17.43 |
| Reading 2 only | 115 | 3.13 | 0.85 | **<0.001** | 1.45 | 4.81 | -14.66 | 20.92 |
| ***DBP: Omron 907 - Omron 705*** |  |  |  |  |  |  |  |  |
| Primary, excluding misordered | 113 | 1.46 | 0.51 | **0.005** | 0.45 | 2.47 | -9.16 | 12.08 |
| Primary, excluding outliers | 114 | 1.52 | 0.50 | **0.003** | 0.52 | 2.52 | -9.05 | 12.08 |
| Mean of 3 readings | 115 | 1.49 | 0.52 | **0.005** | 0.47 | 2.51 | -9.05 | 12.08 |
| Reading 2 only | 115 | 1.55 | 0.67 | **<0.001** | 0.22 | 2.87 | -12.52 | 15.62 |
| **Grip strength, kg** |  |  |  |  |  |  |  |  |
| ***Jamar Hydraulic - Smedley*** |  |  |  |  |  |  |  |  |
| Primary, excluding outliers | 116 | -0.06 | 0.48 | 0.893 | -1.02 | 0.89 | -10.20 | 10.07 |
| Mean of 4 readings | 118 | 0.63 | 0.47 | 0.183 | -0.30 | 1.57 | -9.43 | 10.70 |
| ***Nottingham -*** ***Jamar Plus+*** |  |  |  |  |  |  |  |  |
| Primary, excluding misordered | 117 | 0.33 | 0.59 | 0.579 | -0.83 | 1.49 | -12.10 | 12.75 |
| Mean of 4 readings | 118 | -0.12 | 0.55 | 0.832 | -1.21 | 0.98 | -11.86 | 11.62 |
| ***Jamar Plus+ - Jamar Hydraulic*** |  |  |  |  |  |  |  |  |
| Primary, excluding misordered | 116 | 4.44 | 0.31 | **<0.001** | 3.83 | 5.05 | -2.08 | 10.95 |
| Primary, excluding outliers | 117 | 4.57 | 0.28 | **<0.001** | 4.01 | 5.13 | -1.41 | 10.55 |
| Mean of 4 readings | 118 | 4.13 | 0.27 | **<0.001** | 3.60 | 4.65 | -1.54 | 9.79 |
| ***Jamar Plus+ - Smedley*** |  |  |  |  |  |  |  |  |
| Primary, excluding outliers | 117 | 4.48 | 0.48 | **<0.001** | 3.53 | 5.43 | -5.65 | 14.61 |
| Mean of 4 readings | 118 | 4.76 | 0.49 | **<0.001** | 3.80 | 5.72 | -5.60 | 15.12 |
| ***Nottingham - Jamar Hydraulic*** |  |  |  |  |  |  |  |  |
| Primary, excluding misordered | 117 | 4.89 | 0.58 | **<0.001** | 3.75 | 6.03 | -7.32 | 17.10 |
| Primary, excluding outliers | 117 | 4.84 | 0.59 | **<0.001** | 3.68 | 6.00 | -7.61 | 17.29 |
| Mean of 4 readings | 118 | 4.01 | 0.55 | **<0.001** | 2.91 | 5.10 | -7.76 | 15.78 |
| ***Nottingham – Smedley*** |  |  |  |  |  |  |  |  |
| Primary, excluding misordered | 117 | 4.92 | 0.73 | **<0.001** | 3.46 | 6.37 | -10.64 | 20.48 |
| Primary, excluding outliers | 117 | 4.79 | 0.71 | **<0.001** | 3.38 | 6.21 | -10.35 | 19.94 |
| Mean of 4 readings | 118 | 4.64 | 0.65 | **<0.001** | 3.35 | 5.93 | -9.22 | 18.50 |
| **Lung function, litres** |  |  |  |  |  |  |  |  |
| ***FEV_1_: Micro Plus - Easy on-PC*** |  |  |  |  |  |  |  |  |
| Primary, excluding misordered | 73 | 0.00 | 0.02 | 0.875 | -0.03 | 0.03 | -0.26 | 0.25 |
| All cases, including C-E | 106 | 0.01 | 0.02 | 0.561 | -0.03 | 0.05 | -0.35 | 0.37 |
| ***FVC: Micro Plus - Easy on-PC*** |  |  |  |  |  |  |  |  |
| Primary, excluding misordered | 66 | -0.47 | 0.03 | **<0.001** | -0.53 | -0.42 | -0.92 | -0.02 |
| All cases, including C-E | 106 | -0.45 | 0.03 | **<0.001** | -0.51 | -0.40 | -1.01 | 0.11 |

SE=standard error; CI=confidence interval; LOA=Limits of Agreement
