# Supplementary material for: Comparison of devices used to measure blood pressure, grip strength and lung function: A randomised cross-over study
Source: PLoS One. 2023 Dec 27;18(12):e0289052. doi: 10.1371/journal.pone.0289052 (PMC10752545; doi:10.1371/journal.pone.0289052)
Supplement: S5 Table — (DOCX) [file pone.0289052.s005.docx]

S5 Table: Sensitivity analysis of order effects for all measures

|  | Order of device | | Independent t-test | | | 95% CI | |
| --- | --- | --- | --- | --- | --- | --- | --- |
|  | 1st | 2nd | Diff | SE | p-value | Lower | Upper |
| ***Blood pressure, mm Hg*** |  |  |  |  |  |  |  |
| ***SBP: Omron 907 - Omron 705*** | O. 705 | O. 907 |  |  |  |  |  |
| Primary, excluding misordered | 55 | 58 | -1.91 | 1.36 | 0.162 | -4.60 | 0.78 |
| Primary, excluding outliers | 56 | 58 | -2.93 | 1.23 | **0.019** | -5.36 | -0.49 |
| Mean of 3 readings | 56 | 59 | -2.74 | 1.27 | **0.032** | -5.25 | -0.24 |
| Reading 2 only | 56 | 59 | -2.48 | 1.68 | 0.144 | -5.82 | 0.86 |
| ***DBP: Omron 907 - Omron 705*** | O. 705 | O. 907 |  |  |  |  |  |
| Primary, excluding misordered | 55 | 58 | 0.27 | 1.02 | 0.791 | -1.76 | 2.30 |
| Primary, excluding outliers | 56 | 58 | -0.49 | 1.01 | 0.629 | -2.50 | 1.52 |
| Mean of 3 readings | 56 | 59 | -1.49 | 1.03 | 0.150 | -3.53 | 0.55 |
| Reading 2 only | 56 | 59 | -1.62 | 1.34 | 0.227 | -4.27 | 1.02 |
| ***Grip strength, kg*** |  |  |  |  |  |  |  |
| ***Jamar Hydraulic - Smedley*** | JH | SM |  |  |  |  |  |
| Primary, excluding outliers | 59 | 57 | -0.59 | 0.96 | 0.539 | -2.50 | 1.31 |
| Mean of 4 readings | 59 | 59 | -1.14 | 0.94 | 0.229 | -3.01 | 0.73 |
| ***Nottingham -*** ***Jamar Plus+*** | NO | JP |  |  |  |  |  |
| Primary, excluding misordered | 57 | 60 | -1.86 | 1.16 | 0.113 | -4.17 | 0.45 |
| Mean of 4 readings | 58 | 60 | -2.15 | 1.09 | 0.051 | -4.31 | 0.01 |
| ***Jamar Plus+ - Jamar Hydraulic*** | JP | JH |  |  |  |  |  |
| Primary, excluding misordered | 59 | 57 | -1.24 | 0.61 | **0.044** | -2.44 | -0.03 |
| Primary, excluding outliers | 60 | 57 | -1.43 | 0.55 | **0.011** | -2.52 | -0.34 |
| Mean of 4 readings | 60 | 58 | -1.22 | 0.52 | **0.021** | -2.25 | -0.18 |
| ***Jamar Plus+ - Smedley*** | JP | SM |  |  |  |  |  |
| Primary, excluding outliers | 59 | 59 | 0.03 | 1.03 | 0.980 | -2.02 | 2.07 |
| Mean of 4 readings | 58 | 59 | -0.38 | 0.96 | 0.692 | -2.28 | 1.52 |
| ***Nottingham - Jamar Hydraulic*** | NO | JH |  |  |  |  |  |
| Primary, excluding misordered | 57 | 60 | -2.09 | 1.14 | 0.069 | -4.35 | 0.17 |
| Primary, excluding outliers | 57 | 60 | -2.00 | 1.16 | 0.089 | -4.30 | 0.31 |
| Mean of 4 readings | 57 | 61 | -2.49 | 1.09 | **0.024** | -4.64 | -0.34 |
| ***Nottingham – Smedley*** | NO | SM |  |  |  |  |  |
| Primary, excluding misordered | 60 | 57 | -3.00 | 1.45 | **0.040** | -5.87 | -0.14 |
| Primary, excluding outliers | 59 | 58 | -3.45 | 1.40 | **0.015** | -6.22 | -0.68 |
| Mean of 4 readings | 60 | 58 | -3.02 | 1.28 | **0.020** | -5.55 | -0.48 |
| ***Lung function, litres*** |  |  |  |  |  |  |  |
| ***FEV_1_: Micro Plus - Easy on-PC*** | MM | Easy |  |  |  |  |  |
| Primary, excluding misordered | 39 | 34 | -0.02 | 0.02 | 0.528 | -0.08 | 0.04 |
| All cases, including C-E | 54 | 52 | -0.01 | 0.04 | 0.795 | -0.08 | 0.06 |
| ***FVC: Micro Plus - Easy on-PC*** | MM | Easy |  |  |  |  |  |
| Primary, excluding misordered | 35 | 31 | -0.06 | 0.06 | 0.287 | -0.17 | 0.05 |
| All cases, including C-E | 54 | 52 | -0.11 | 0.05 | 0.053 | -0.22 | 0.00 |

SE=standard error; CI=confidence interval
