# Supplementary material for: Comparison of devices used to measure blood pressure, grip strength and lung function: A randomised cross-over study
Source: PLoS One. 2023 Dec 27;18(12):e0289052. doi: 10.1371/journal.pone.0289052 (PMC10752545; doi:10.1371/journal.pone.0289052)
Supplement: S6 Table — (DOCX) [file pone.0289052.s006.docx]

S6 Table: Sensitivity analysis using multilevel models for blood pressure

| Omron 907 - Omron 705 |  | Paired t-test | | | 95% CI | |
| --- | --- | --- | --- | --- | --- | --- |
| Systolic blood pressure, mm Hg | N | Diff | SE | p-value | Lower | Upper |
| Based on mean of 2^nd^+3^rd^ | 115 | 3.86 | 0.69 | <0.001 | 2.50 | 5.22 |
| Based on mean of 3 readings | 115 | 3.92 | 0.64 | <0.001 | 2.65 | 5.19 |
| - M1: device only | 689* | 3.93 | 0.46 | <0.001 | 3.02 | 4.83 |
| - M2: Order, dom, hand, seq | 689* | 3.89 | 0.46 | <0.001 | 3.00 | 4.79 |
| - M3: M2 + demographics | 683* | 3.91 | 0.46 | <0.001 | 3.00 | 4.81 |
| Diastolic blood pressure, mm Hg | N | Diff | SE | p-value | Lower | Upper |
| Based on mean of 2^nd^+3^rd^ | 115 | 1.35 | 0.53 | 0.012 | 0.30 | 2.39 |
| Based on mean of 3 readings | 115 | 1.49 | 0.52 | 0.005 | 0.47 | 2.51 |
| - M1: device only | 689* | 1.53 | 0.32 | <0.001 | 0.91 | 2.16 |
| - M2: Order, dom, hand, seq | 689* | 1.51 | 0.31 | <0.001 | 0.90 | 2.13 |
| - M3: M2 + demographics | 683* | 1.56 | 0.31 | <0.001 | 0.95 | 2.18 |

Shaded rows are results from primary analyses and the sensitivity analysis based on the mean of 3 readings for comparison with results from multilevel models which include.

*number of measurements (up to 6 per individual)

M1 includes only device

M2 included order of device, whether hand was dominant, left or right and sequence of reading

M3 additional included age, sex, BMI

N is the number of BP observations included in analyses

SE=standard error; CI=confidence interval
