# Supplementary material for: Comparison of devices used to measure blood pressure, grip strength and lung function: A randomised cross-over study
Source: PLoS One. 2023 Dec 27;18(12):e0289052. doi: 10.1371/journal.pone.0289052 (PMC10752545; doi:10.1371/journal.pone.0289052)
Supplement: S7 Table — (DOCX) [file pone.0289052.s007.docx]

S7 Table: Sensitivity analysis using multilevel models for grip strength

| Omron 907 - Omron 705 |  | Paired t-test | | | 95% CI | |
| --- | --- | --- | --- | --- | --- | --- |
| Jamar Hydraulic – Smedley | N | Diff | SE | p-value | Lower | Upper |
| Based on maximum | 118 | 0.23 | 0.52 | 0.654 | -0.79 | 1.26 |
| Based on mean of 4 readings | 118 | 0.63 | 0.47 | 0.183 | -0.30 | 1.57 |
| - M1: 4 separate readings | 472* | 0.63 | 0.30 | **0.037** | 0.04 | 1.23 |
| - M2: Order, seq, dom, hand | 472* | 0.64 | 0.28 | **0.023** | 0.09 | 1.20 |
| - M3: M2 and demographics | 472* | 0.64 | 0.28 | **0.023** | 0.09 | 1.20 |
| Nottingham - Jamar Plus+ |  |  |  |  |  |  |
| Based on maximum | 118 | 0.29 | 0.58 | 0.623 | 0.87 | 1.44 |
| Based on mean of 4 readings | 118 | -0.12 | 0.55 | 0.832 | -1.21 | 0.98 |
| - M1: 4 separate readings | 472* | 0.16 | 0.30 | 0.595 | -0.43 | 0.75 |
| - M2: Order, seq, dom, hand | 472* | 0.20 | 0.28 | 0.484 | -0.36 | 0.75 |
| - M3: M2 and demographics | 472* | 0.20 | 0.28 | 0.484 | -0.36 | 0.75 |
| Jamar Plus+ - Jamar Hydraulic |  |  |  |  |  |  |
| Based on maximum | 118 | 4.45 | 0.30 | **<0.001** | 3.85 | 5.05 |
| Based on mean of 4 readings | 118 | 4.13 | 0.27 | **<0.001** | 3.60 | 4.65 |
| - M1: Separate readings | 472* | 4.13 | 0.30 | **<0.001** | 3.53 | 4.72 |
| - M2: Order, seq, dom, hand | 472* | 4.15 | 0.28 | **<0.001** | 3.60 | 4.71 |
| - M3: M2 and demographics | 472* | 4.15 | 0.28 | **<0.001** | 3.60 | 4.71 |
| Jamar Plus+ - Smedley |  |  |  |  |  |  |
| Based on maximum | 118 | 4.68 | 0.52 | **<0.001** | 3.66 | 5.70 |
| Based on mean of 4 readings | 118 | 4.76 | 0.49 | **<0.001** | 3.80 | 5.72 |
| - M1: Separate readings | 472* | 4.76 | 0.30 | **<0.001** | 4.17 | 5.35 |
| - M2: Order, seq, dom, hand | 472* | 4.80 | 0.28 | **<0.001** | 4.24 | 5.35 |
| - M3: M2 and demographics | 472* | 4.80 | 0.28 | **<0.001** | 4.24 | 5.35 |
| Nottingham - Jamar Hydraulic |  |  |  |  |  |  |
| Based on maximum | 118 | 4.74 | 0.59 | **<0.001** | 3.57 | 5.91 |
| Based on mean of 4 readings | 118 | 4.01 | 0.55 | **<0.001** | 2.91 | 5.10 |
| - M1: 4 separate readings | 472 | 3.97 | 0.30 | **<0.001** | 3.37 | 4.56 |
| - M2: Order, seq, dom, hand | 472 | 3.95 | 0.28 | **<0.001** | 3.40 | 4.51 |
| - M3: M2 and demographics | 472 | 3.95 | 0.28 | **<0.001** | 3.40 | 4.51 |
| Nottingham – Smedley |  |  |  |  |  |  |
| Based on maximum | 118 | 4.97 | 0.73 | **<0.001** | 3.52 | 6.41 |
| Based on mean of 4 readings | 118 | 4.64 | 0.65 | **<0.001** | 3.35 | 5.93 |
| - M1: 4 separate readings | 472 | 4.60 | 0.30 | **<0.001** | 4.01 | 5.19 |
| - M2: Order, seq, dom, hand | 472 | 4.60 | 0.28 | **<0.001** | 4.04 | 5.16 |
| - M3: M2 and demographics | 472 | 4.60 | 0.28 | **<0.001** | 4.04 | 5.16 |

Shaded rows are results from primary analyses and the sensitivity analysis based on the mean of 4 readings for comparison with results from multilevel models.

*number of measurements (up to 4 per individual)

M1 includes only device

M2 included order of device, whether hand was dominant, left or right and sequence of reading

M3 additional included age, sex, BMI

N is the number of grip strength observations included in analyses

SE=standard error; CI=confidence interval
