# Supplementary material for: Comparison of devices used to measure blood pressure, grip strength and lung function: A randomised cross-over study
Source: PLoS One. 2023 Dec 27;18(12):e0289052. doi: 10.1371/journal.pone.0289052 (PMC10752545; doi:10.1371/journal.pone.0289052)
Supplement: S8 Table — (DOCX) [file pone.0289052.s008.docx]

S8 Table: Sensitivity analysis using multilevel models for lung function

|  |  | Paired t-test | | | 95% CI | |
| --- | --- | --- | --- | --- | --- | --- |
| FEV_1_, litres | N | Diff | SE | p-value | Lower | Upper |
| Primary analysis |  |  |  |  |  |  |
| Micro Plus - Easy on-PC | 74 | -0.002 | 0.001 | 0.880 | -0.032 | 0.028 |
| Sensitivity analysis |  |  |  |  |  |  |
| Primary, excluding misordered | 73 | -0.002 | 0.015 | 0.875 | -0.033 | 0.028 |
| All cases, including quality C-E | 106 | -0.010 | 0.018 | 0.561 | -0.025 | 0.046 |
| Multilevel models |  |  |  |  |  |  |
| - M1: all readings | 707* | -0.012 | 0.012 | 0.297 | -0.036 | 0.011 |
| - M2: sequence | 707* | -0.014 | 0.012 | 0.252 | -0.037 | 0.010 |
| - M3: M2+demog+health | 707* | -0.014 | 0.012 | 0.249 | -0.037 | 0.010 |
| FVC, litres | N | Diff | SE | p-value | Lower | Upper |
| Primary analysis, FVC |  |  |  |  |  |  |
| Micro Plus - Easy on-PC | 67 | -0.474 | 0.028 | <0.001 | -0.530 | -0.418 |
| Sensitivity analysis, FVC |  |  |  |  |  |  |
| Primary, excluding misordered | 66 | -0.472 | 0.028 | <0.001 | -0.529 | -0.416 |
| All cases, including quality C-E | 106 | -0.452 | 0.028 | <0.001 | -0.507 | -0.397 |
| Multilevel models, FVC |  |  |  |  |  |  |
| - M1: all readings | 705* | -0.446 | 0.015 | <0.001 | -0.474 | -0.417 |
| - M2: sequence | 705* | -0.445 | 0.015 | <0.001 | -0.474 | -0.416 |
| - M3: M2+demog+health | 705* | -0.445 | 0.015 | <0.001 | -0.474 | -0.416 |

Shaded rows are results from primary analyses.

*number of measurements (up to 10 per individual)

M1 includes only device

M2 included order of device, whether hand was dominant, left or right and sequence of reading

M3 additional included age, sex, BMI

N is the number of grip strength observations included in analyses

SE=standard error; CI=confidence interval; LOA=Limits of agreement
