# Supplementary material for: Comparison of devices used to measure blood pressure, grip strength and lung function: A randomised cross-over study
Source: PLoS One. 2023 Dec 27;18(12):e0289052. doi: 10.1371/journal.pone.0289052 (PMC10752545; doi:10.1371/journal.pone.0289052)
Supplement: S1 Fig — (DOCX) [file pone.0289052.s009.docx]

# S1 Fig. Histograms of mean differences in SBP (mmHg), DBP (mmHg), maximum grip strength (kg) and lung function (FEV_1_ and FVC, litres) for all device combinations

| SBP: Omron 705 - 905 (Mean 2^nd^+3^rd^)  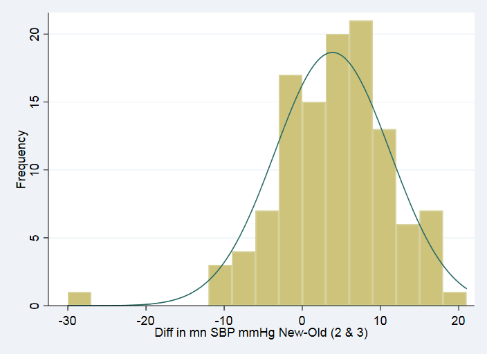 | DBP: Omron 705 - 905 (Mean 2^nd^+3^rd^)  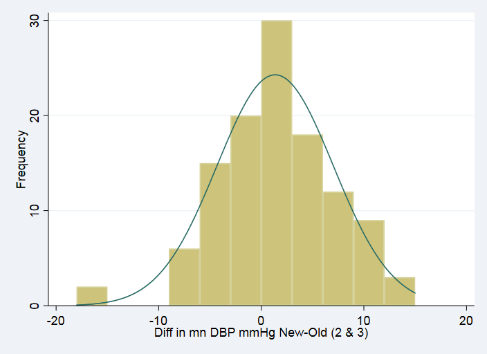 |
| --- | --- |
| Nottingham - Jamar Plus+ (max of 4)  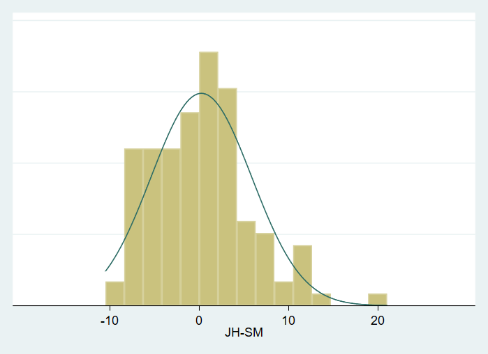 | Jamar Hydraulic - Smedley (max of 4)  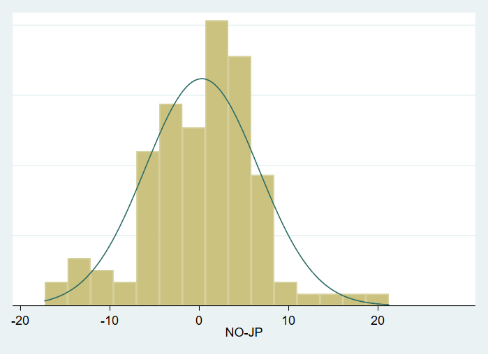 |
| Jamar Plus+ - Jamar Hydraulic (max of 4)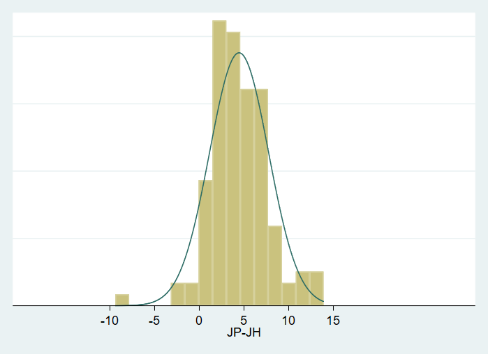 | Jamar Plus+ - Smedley (max of 4)  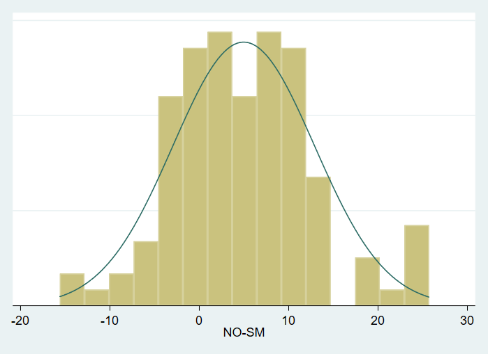 |
| Nottingham - Jamar Hydraulic (max of 4)  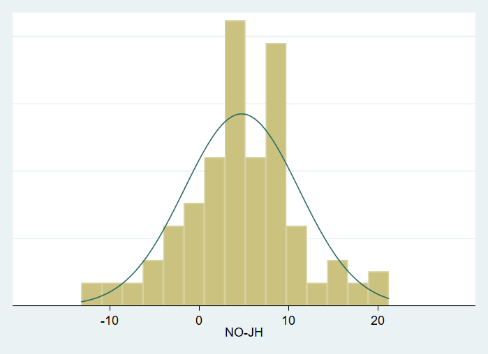 | Nottingham - Smedley (max of 4)  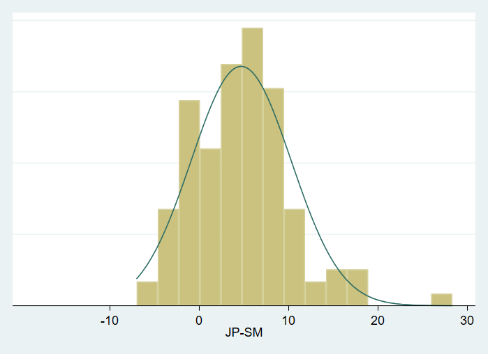 |
| FEV_1_: Micro Plus-Easy on-PC (A&B)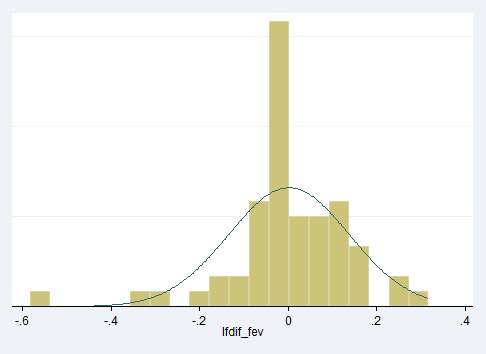 | FVC: Micro Plus - Easy on-PC (A&B)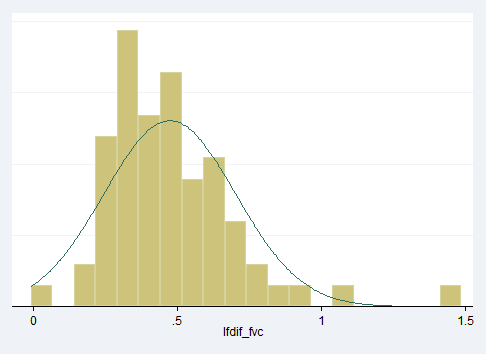 |
