# Supplementary material for: Comparison of devices used to measure blood pressure, grip strength and lung function: A randomised cross-over study
Source: PLoS One. 2023 Dec 27;18(12):e0289052. doi: 10.1371/journal.pone.0289052 (PMC10752545; doi:10.1371/journal.pone.0289052)
Supplement: S1 Appendix — (DOCX) [file pone.0289052.s010.docx]

Supplementary methods

**Sample size calculations**

The sample size was calculated to ensure sufficient statistical power to be able to identify differences of an expected scale between devices. This was based on the assumption that the correlation between the measurements of the instruments would be higher than 0.8 (Kaaks R, 1995).

**Sample source**

The sample was selected from individuals who had taken part in the TNS Omnibus survey. The Omnibus is based on a multi-phase sampling design in which the geography of Great Britain was stratified using 2011 Census small area statistics and the Postcode Address File to define sample points. Clusters of wards were selected within these sample points and within these clusters Census Output Areas were sampled. Participants were recruited within these areas, meeting a set of quotas.

Individuals who responded to the opening section of the TNS Omnibus interview were asked if they were willing to be re-contacted for future research purposes. If they agreed, they were added to the “Demographic Database”. The sample for the machine comparison study was drawn from individuals in the Demographic Database in London and the South East.

**Eligibility criteria**

Exclusion criteria were: a chest infection (such as influenza, pneumonia, bronchitis, severe cold) in the last 4 weeks; coughing up blood of unknown origin in the last 4 weeks; a heart attack or other heart complaint in the last 6 weeks; a stroke in the last 6 weeks; abdominal or chest surgery in the last 3 months; ever having been diagnosed with an aneurysm in chest, brain or stomach; a detached retina or eye surgery in the last 3 months; ear surgery in the last 3 months; a collapsed or punctured lung in the last 12 months; a blood clot in the lung in the last 3 months and; currently on medication for tuberculosis.

In addition, before the grip strength assessment took place, participants were excluded if they had severely raised blood pressure (systolic ≥200mmHg or diastolic ≥120mm Hg) or if either hand could not be assessed because of swelling or inflammation, severe pain or recent injury, or hand surgery in the last six months. Neither of these situations arose.
