# Supplementary material for: Comparison of devices used to measure blood pressure, grip strength and lung function: A randomised cross-over study
Source: PLoS One. 2023 Dec 27;18(12):e0289052. doi: 10.1371/journal.pone.0289052 (PMC10752545; doi:10.1371/journal.pone.0289052)
Supplement: Attachment — Submitted filename: Review_PONE-D-22-31125.docx [file pone.0289052.s012.docx]

**Review of PONE-D-22-31125**

Thank you for the invitation to review the manuscript “Comparison of devices used to measure blood pressure, grip strength and lung function: a randomised cross-over study”.

The manuscript is well-written, and the research question is clearly defined. The study is scientifically sound, and I have no significant concerns. I therefore provide only minor comments and suggestions which I hope the authors will find useful in improving the presentation of their work.

**Abstract**

It would be useful to state that multiple measurements were taken for each device.

L58: consider providing quantitative estimates with the statement that “differences were small”, for example the range of mean differences.

Key implications of the findings for statistical analyses could be more specific, addressing how these findings should be considered when “modelling intra-individual changes in function and when carrying out cross-study analyses.”

**Introduction**

A brief paragraph discussing the test-retest reliability of these devices would be useful to contextualise the findings regarding difference between devices. How do measurements taken with the same device vary in magnitude compared to differences between devices?

L89-91: could these differences instead be accounted for in the analyses? It would be useful if the authors would be more specific about what they mean by “in some instances”.

L94: although the authors cite multiple studies, they state that the “evidence is limited”.

L98 (and elsewhere): I suggest avoiding unnecessary acronyms such as “BP” for blood pressure.

**Methods**

L104: consider providing the name of the guidelines (CONSORT) here.

L109: “and the South East” of England.

L119: “anonymised” -> “pseudo anonymised”.

Please provide the justification/rationale for using a randomised cross-over study design. This might seem obvious, but it is useful to state this explicitly in the text.

As noted above, it would be useful to state earlier that multiple measurements were taken with each device.

Data on other standard metrics for lung function (e.g., peak expiratory flow or the ratio of FEV_1_/FVC) were not considered here and might be worth adding, if available.

More details on quality control should be provided for the lung function data.

L210-211: additional guidance should be provided for readers regarding the interpretation of 95% limits of agreement.

**Results**

Table 3: “V good” and “V poor” should be spelled out.

Table 4: “Some/lot difficulting gripping” needs revision.

The data on reliability provided in Table S2 are of interest. Could the authors provide information about what these reliability estimates translate to in terms of variation in units of measurement? This would likely help readers better interpret the results regarding between-device differences.

L275: “… the analyses”.

The large differences between digital dynamometers and manual ones are of interest. Did these discrepancies vary by experimenter?

**Discussion**

L340: please explain why the age of the sample would be a relevant consideration here.

L345: “55 participants aged …” would make for easier reading.

L364: given the strong non-linear associations between age and several of these measures (e.g., Mutz et al. 2021, Aging, doi: 10.18632/aging.203275 in a large UK sample), it is perhaps difficult to talk about the equivalent of a 5-year age difference, especially for measures like diastolic blood pressure.

L405: although this suggestion is appropriate within studies, I am not sure about the feasibility of conducting such experiments when making comparison between studies (which are typically secondary data analyses / meta-analyses).

L426: “irrepsctive” -> “irrespective”.

Could the authors comment on how statistically significant differences, for example, between groups should be interpreted if such differences are smaller in magnitude than some of the differences observed here between devices for the same device across multiple measurements?
