# Supplementary material for: Comparison of devices used to measure blood pressure, grip strength and lung function: A randomised cross-over study
Source: PLoS One. 2023 Dec 27;18(12):e0289052. doi: 10.1371/journal.pone.0289052 (PMC10752545; doi:10.1371/journal.pone.0289052)
Supplement: Attachment — Submitted filename: Response to reviewers.docx [file pone.0289052.s013.docx]

**Response to reviewers**

Reviewer #1

Carli Lessof et al. used a randomized cross-over study to compare different devices for their measurement of blood pressure, grip strength and lung function. All the measurements and analyses were performed to a good technical standard. Measurement differences between devices are bound to exist, however, the article does not provide information for us to choose a better device. I recommend comparing these devices to standard devices such as mercury sphygmomanometer.

**Response**: We thank the reviewer for acknowledging that our study was performed to a good technical standard. The intention of the study was not to aid selection of a “better device” nor to compare new devices with a standard machine, but rather to aid within- and between-study comparison of devices already commonly used in longitudinal studies. We therefore selected devices which had commonly been used in key UK longitudinal population studies for which there was a lack of previous comparison information. There have been multiple previous studies comparing the manual sphygmomanometers, including mercury ones, with automated devices (see references 16-20), but few comparing different automated devices. We have made edits to the introduction to make clearer the rationale for the choice of machines compared in our study (lines 81-104).

Reviewer #2:

The manuscript is well-written, and the research question is clearly defined. The study is scientifically sound, and I have no significant concerns. I therefore provide only minor comments and suggestions which I hope the authors will find useful in improving the presentation of their work.

**Response**: We thank the reviewer for their positive comments and for the very helpful suggestions which we feel have improved the manuscript.

Abstract

It would be useful to state that multiple measurements were taken for each device.

**Response**: This information has been added to the abstract (line 49).

L58: consider providing quantitative estimates with the statement that “differences were small”, for example the range of mean differences.

**Response**: The estimates and 95% confidence intervals have been added (lines 59 and 60).

Key implications of the findings for statistical analyses could be more specific, addressing how these findings should be considered when “modelling intra-individual changes in function and when carrying out cross-study analyses.”

**Response**: The implications have been edited to make this clearer and highlight that we might expect sensitivity analyses to be carried out (lines 66-67).

Introduction

A brief paragraph discussing the test-retest reliability of these devices would be useful to contextualise the findings regarding difference between devices. How do measurements taken with the same device vary in magnitude compared to differences between devices?

**Response**: On review of the literature, there was little good quality information on test-retest reliability across the devices used in this study. We therefore decided not to include a paragraph in the introduction, but we acknowledge that this is an important point to consider. Therefore, in response to this point, and others below, we have added the within-person standard deviations to Table S2 (and discuss in lines 254-260) and further discussion of reliability in interpretation of the findings (lines 391-394, 406-407, 413-416, 430-435).

L89-91: could these differences instead be accounted for in the analyses? It would be useful if the authors would be more specific about what they mean by “in some instances”.

**Response**: We have clarified this sentence in that it relates to specific recommendations regarding the use of spirometers in UK studies (line 94).

L94: although the authors cite multiple studies, they state that the “evidence is limited”.

**Response**: We have edited the final paragraph of the introduction to make clearer our choice of machines to compare (lines 97-104).

L98 (and elsewhere): I suggest avoiding unnecessary acronyms such as “BP” for blood pressure.

**Response**: We have used blood pressure instead of BP and also replace LOA with limits of agreement and BMI with body mass index. We welcome editorial guidance on any other acronyms that should be replaced.

Methods

L104: consider providing the name of the guidelines (CONSORT) here.

**Response:** “CONSORT” has been added (line 110).

L109: “and the South East” of England.

**Response**: This has been corrected (line 115).

L119: “anonymised” -> “pseudo anonymised”.

**Response**: This had been corrected (line 125).

Please provide the justification/rationale for using a randomised cross-over study design. This might seem obvious, but it is useful to state this explicitly in the text.

**Response**: We thank the reviewer for highlighting the need for clarification. The rationale for using a randomised cross-over study design has now been added. “…so as to make within-person measurement comparisons” (lines 109).

As noted above, it would be useful to state earlier that multiple measurements were taken with each device.

**Response:** This has now been stated in the “Study Design and Sample” section (lines 135-136).

Data on other standard metrics for lung function (e.g., peak expiratory flow or the ratio of FEV1/FVC) were not considered here and might be worth adding, if available.

**Response**: FEV1 and FVC are the gold standard measures for lung function and therefore are the ones that are derived from spirometers in longitudinal population studies and used in analyses. PEFR is seen as an approximation of FEV1, and is therefore no longer used where FEV1 measures are available. FEV1 and FVC were thus chosen *a priori* as primary outcomes measures. Given these measures were defined in our protocol, we feel that it would not be good practice to add PEFR as an outcome at this stage and, although available, our protocol was not set up to standardise PEFR measurement. We do acknowledge the interest in the FEV1/FVC particularly in detecting COPD, and we have thus added a sentence to the discussion on the likely impact of our findings on FEV1/FVC (lines 418-419). Given that this outcome is a ratio of two measurements, a comparison between devices would not be informative as differences would be a combination of the variation in the individual components. If adjustment for a change in device was to be made, it would be to the component measures (FEV1 or FVC) rather than the ratio.

More details on quality control should be provided for the lung function data.

**Response**: We have added details on the quality control (lines 201-203). In relation to this, we also clarified that participants had five attempts to produce three valid measures (lines 179-180).

L210-211: additional guidance should be provided for readers regarding the interpretation of 95% limits of agreement.

**Response**: This has been added (lines 220-221), as has a clearer description of the Bland-Altman plot which also aids interpretation (lines 217-218).

Results

Table 3: “V good” and “V poor” should be spelled out.

**Response**: These have been spelled out.

Table 4: “Some/lot difficulting gripping” needs revision.

**Response**: This has been edited*.*

The data on reliability provided in Table S2 are of interest. Could the authors provide information about what these reliability estimates translate to in terms of variation in units of measurement? This would likely help readers better interpret the results regarding between-device differences.

**Response**: We have added the within-person standard deviation, which are in the original units, for each device and measurement to Table S2. We have added additional text describing the content of this table, including highlighting the repeatability is part of the spirometry quality control (lines 254-260).

L275: “… the analyses”.

**Response:** This has been corrected.

The large differences between digital dynamometers and manual ones are of interest. Did these discrepancies vary by experimenter?

**Response**: Given that this is a within-person comparison study, and the same experimenter (assessor) tested the same person on all machines, while the individual measurements may have varied according to experimenter (due, for example, to different levels of encouragement), we did not anticipate that they would have had a great impact on the differences. However, we did consider the potential impact of assessor variation in preliminary multilevel model analyses. These models showed that the variation by assessor, as anticipated, was small (and was statistically non-significant), and inclusion of this source of variation did not change the main findings. Therefore, we chose not include assessor in our final analyses and can conclude therefore that assessor does not explain the wide variation in differences in grip strength. We do now acknowledge that assessor is an additional source of variation which we have not accounted for in the study and have added this to the discussion (lines 448-449).

Discussion

L340: please explain why the age of the sample would be a relevant consideration here.

**Response:** This has been explained. “However, the study population was younger, with an average age of 32 years, comprising a convenience sample of 40 men and women and may have better function than our older sample which could influence comparability across machines” (lines 361-362)

L345: “55 participants aged …” would make for easier reading.

**Response**: This has been changed (line 366)

L364: given the strong non-linear associations between age and several of these measures (e.g., Mutz et al. 2021, Aging, doi: 10.18632/aging.203275 in a large UK sample), it is perhaps difficult to talk about the equivalent of a 5-year age difference, especially for measures like diastolic blood pressure.

**Response**: we have added a caveat to this interpretation for diastolic blood pressure and acknowledge the limitation of this comparison (line 404-406).

L405: although this suggestion is appropriate within studies, I am not sure about the feasibility of conducting such experiments when making comparison between studies (which are typically secondary data analyses / meta-analyses).

**Response**: This point been edited for clarity. “Conducting external comparison studies, such as ours, would also help interpretation for both within-study and between-study comparisons.” (lines 429-430)

L426: “irrepsctive” -> “irrespective”.

**Response**: This has been corrected.

Could the authors comment on how statistically significant differences, for example, between groups should be interpreted if such differences are smaller in magnitude than some of the differences observed here between devices for the same device across multiple measurements?

**Response**: As indicated above we have added the within-person standard deviations in Table S2. We have also edited the discussion to add further comment on the interpretation (lines 391-394, 406-407, 413-416, 430-435).
