# Supplementary material for: Comparison of devices used to measure blood pressure, grip strength and lung function: A randomised cross-over study
Source: PLoS One. 2023 Dec 27;18(12):e0289052. doi: 10.1371/journal.pone.0289052 (PMC10752545; doi:10.1371/journal.pone.0289052)
Supplement: Attachment — Submitted filename: Response to reviewers2.docx [file pone.0289052.s014.docx]

**Response to additional reviewers comments from 23/05/2023**

Reviewer #1: The study showed measurement differences between devices commonly used to assess BP, grip strength and lung function, the results may help within and between-study comparison of devices used in longitudinal studies. I only provide minor suggestions which I hope the authors will find useful in improving the presentation of their work

1. I suggest presenting the order of assessment (Table 2) in the form of a flowchart.

**Response:** Thank you for this suggestion. We have provided a flowchart.

2. Please add the limitations of this study in the Discussion.

**Response:** We have made the limitations of this study more explicit and expanded the discussion.
